# Nutrition improvement program for rural compulsory education students and individual health

**DOI:** 10.3389/fpubh.2022.1051810

**Published:** 2022-11-08

**Authors:** Yinhe Liang, Xiyin Chen, Chenxiao Zhao, Shaoxiang Jiang

**Affiliations:** ^1^School of Economics, Central University of Finance and Economics, Beijing, China; ^2^Department of International Health, Johns Hopkins Bloomberg School of Public Health, Baltimore, MD, United States; ^3^China Export and Credit Insurance Corporation, Beijing, China; ^4^Institute for Global Health and Development, Peking University, Beijing, China; ^5^National School of Development, Peking University, Beijing, China

**Keywords:** nutrition improvement program, adolescent health, health services and outcomes research, children health, rural compulsory education

## Abstract

We use the county-by-county rollout of the program and employ the difference-in-difference (DID) methodology to identify the effects of the implementation of the nutrition improvement program for rural compulsory education students on adolescent health. The results show that the nutrition improvement program reduces the frequency and probability of illness and improves the students' health status. The heterogeneity analysis indicates that the children in western regions and children left behind have a greater marginal improvement. It finds that the nutrition improvement program for rural compulsory education students improves adolescent health through diversifying nutrition intake to alleviate malnutrition and developmental delay in impoverished areas. The program can promote adolescent health in impoverished areas, which has a role in improving regional health disparities and alleviating the intergenerational entrenchment of poverty.

## Introduction

The existing literature finds that children's health status is related to their early human capital formation and their income, health, and creative capacity in adulthood (1, 2)^1^. Despite this, approximately 66 million schoolchildren in developing countries still attend school while hungry (6), and approximately two-thirds of children do not receive the minimum recommended dietary diversity for healthy growth and development (7). In order to address the issue of school-age child hunger, there are 388 million school-age children currently being fed through school feeding programs in 161 countries worldwide (8). School feeding programs have also become the most extensive social safety net in the world.

In order to improve health status of rural students, Chinese government began to implement a nutrition improvement program for students in rural compulsory education. By 2020, 1,762 counties in 29 provinces in China have implemented the nutrition improvement program, accounting for 84.12% of the total number of rural compulsory education schools and benefiting more than 40 million students. On one hand, this policy is mandatory and free. Compulsorily driven by the government, all students in the stage of compulsory education (7–15 years old) in those implementing areas must participate in the program. Moreover, since the program is funded by the central government, all the students can enjoy nutritious meal service free of charge. On the other hand, the policy has been gradually implemented at the county level since 2011, which helps us to construct a difference-in-differences (DID) model to assess the effectiveness of the policy exploiting the variation in the implementation of the policy at district and time level.

Therefore, we analyze the impact of the nutrition improvement program on individual's health status and its impact mechanism based on differences-in-differences approach using micro panel data from 2010 to 2016 China Family Panel Studies (CFPS). We find that the nutrition improvement program leads to the decrease in the frequency of illness by 0.2 times and the decrease in the probability of illness by about 9%, compared with non-implementing areas. The result is still robust after including income, parents, and district fixed effects. The heterogeneity analysis shows that children in western regions, left-behind children and male children are affected by the policy more significantly. Furthermore, we explore the impact mechanism of the nutrition improvement program and find that the nutrition improvement program improves students' health status by optimizing their dietary structure, making their diets more diversified and promoting their physical quality.

This paper has several appealing features: (i) It extends the literature on student nutrition improvement programs. The existing studies mainly focus on Sweden (9), the United States (10, 11), India (12), Ghana (13)^2^ etc. and studies on the nutrition program in China are deficient. (ii) It extends the literature on students' health. The existing studies mainly focus on parents' migrant work (14)^3^, male preference (15, 16)^4^, physical activity (17), medical insurance and health infrastructure (18)^5^. In contrast to these studies, this paper focuses on the health improvement effects of the student nutrition improvement program. (iii) It deepens the analysis of impact mechanism. The study of mechanism is crucial for refinement of the nutrition improvement program. Therefore, this paper presents the mechanistic analysis of nutrition diversity and physical development, respectively.

## Data and empirical strategy

### Nutrition improvement program for rural compulsory education students

Although the nutritional status of Chinese children has improved significantly over the past three decades, the prevalence of malnutrition among rural children remains prominent. According to the Fifth China National Nutrition and Health Survey in 2012, the prevalence of stunting and underweight among children under 6 years of age in poor rural areas was 19.1 and 5.1%, respectively. In particular, the prevalence of vitamin A deficiency among rural children aged 6–12 years in poor rural areas was 32.9%. Meanwhile, according to the 2010 census data, 61 million children were left behind in rural areas of China, and parental absence further worsened the nutritional status of rural children (19). In order to improve the nutritional status of children in compulsory education in rural areas, the Chinese government has gradually implemented a nutrition improvement program since 2012.

At present, 137,000 schools in 29 provinces in China have implemented the nutrition improvement program, which means that more than half of the compulsory education schools provide nutritious meals to students every day, and nearly a quarter of the compulsory education students can eat nutritious meals at school. The program mainly focuses on the following three parts. First, it provides nutritious meal subsidies for rural compulsory education students. Second, it funds boarding students with financial difficulties living expenses. Third, it carries out the construction of cafeterias, further increases the proportion of meals served in cafeterias, continuously enriches the meals served and environment of cafeterias, and ensures that all school cafeterias are licensed to operate. By the end of 2021, the central government had arranged a total of 196,734 billion yuan for nutritional meal subsidies for students, benefiting more than 30 million students each year, which plays a vital role in students' physical and mental health in impoverished mountainous areas as well as rural areas. Initially, the subsidy standard was 3 yuan per student per day, and then increased to 4 yuan per student per day in November 2014 (calculated according to 200 days of school time in a year). It basically solves the problem of poor students eating at school and reduces the financial burden of poor families.

The implementation of the nutrition improvement program has experienced two main phases. The first phase is from 2012 to 2016. Six hundred and ninety-nine state pilot counties and 616 district pilot counties are included in the program, of which state poverty counties are the main ones, and almost all of them are state or province poverty counties. Also, the list of pilot counties remains unchanged within that period. In the second phase, in December 2016, 10 more provinces and regions expand the scope of the implementation under the promotion of the state, and the total number of pilot counties is expanded to 813. The nutrition improvement program in China has a clear implementation timeline and a list of pilot counties, which provides a good basis for quantitive policy evaluation.

### CFPS data

Our analysis relies on micro panel data collected by China Family Panel Studies (CFPS) in 2010, 2012, 2014, and 2016. CFPS is held by Institute of Social Science Survey (ISSS) of Peking University, which is a crucial social science project aiming at investigating society, economy, population, education and health in China. The CFPS sample covers 25 provinces and cities, with a target sample size of 16,000 households, making it a nationally representative tracking survey. The survey asks detailed questions about household economic behavior, adult and child health performance, and demographic attributes. We exploit CFPS data based on the following three reasons. First, CFPS interviews people under 15 years old separately, which is the population we focus on. Second, panel data enables us to control district-level heterogeneity to obtain robust estimates. Third, since the nutrition improvement program is implemented in 2012, CFPS is one of the few micro panel data that contain population data both before and after 2012.

We further restrict data in the following two ways: (i) We remove youth individuals under 15 years old with 1 year missing from the questionnaire over 4 years to ensure that the data are panel data. (ii) We restrict the analysis samples to those in rural areas. Since the definition of rural areas in the nutrition improvement program for rural compulsory education students is distinguished by district and county, rather than by whether the residence is urban or rural, we conduct our analysis at the district and county level. If the last word of the area name is not “district” or “city” (except for state and province poverty counties), we consider it to be a rural area.

### Empirical strategy

The purpose of this paper is to assess the casual effect of the nutrition improvement program on rural adolescent health. The gradual implementation of student nutrition improvement policies at the district and county levels in China has created the ideal environment for us to adopt the DID approach for identification^6^. Specifically, DID estimation involves comparing child health outcomes of counties before and after they adopted the SNIP with that of counties that had not adopted it during the same period. The model can be written as:


(1) $$\begin{array}{l} health_{ict}=\alpha +\beta *SNIP_{ct}+X_{ict}+\varphi_{c}+\rho_{t}+\epsilon_{ijt} \end{array}$$

where dependent variable *health*_*ict*_ exhibits individual health status, measured by the frequency of illness in the last month, whether they were sick or not in the last month, and self-rating health status, respectively. *SNIP*_*ct*_ is a dummy variable and measures the implementation of the nutrition improvement program, where *SNIP*_*ct*_ = 1 after the policy is implemented and *SNIP*_*ct*_ = 0 before the policy is implemented. Coefficient β exhibits the effect of the nutrition improvement program on individual health. We also control for the following three aspects. First, *X*_*ict*_ denotes individual-level control variables, which are represented by the log of annual household income, gender, age, parents' education level, whether parents live together or not, distance to medical site, availability of medical insurance and water sources for the household. *X*_*ict*_ mainly controls factors that may affect individual health to ensure the validity of estimates. Second, φ_*c*_ denotes district fixed effect and controls all factors at the district level that may affect individual health. Third, ρ_*t*_ denotes year fixed effect and controls all factors at the time level that may affect individual health. The standard errors are clustered by the district-year level.

The key assumption of DID approach is that implementing areas and non-implementing areas satisfy parallel trend assumption. However, pilot counties in 2012 are mainly composed of state and province poverty counties, which indicates that implementing areas and non-implementing areas have significant differences in geographical location, economic development, medical conditions etc. Therefore, Equation (1) may underestimate the result.

To solve the problem above, we restrict the analysis samples to state poverty counties and both state and province poverty counties and regress these two models, respectively. State and province poverty counties are impoverished and internal economy structure can be very similar. Therefore, implementing areas and non-implementing areas which are included in poverty counties can satisfy parallel trend assumption. Moreover, Table 1 shows that 699 districts and counties start to implement the nutrition improvement program in 2012, of which 592 are state poverty counties and 691 are province poverty counties, and there are 392 non-implementing areas within state and province poverty counties in 2012. Table 2 summarizes the descriptive statistics for all the variables used in our analysis.

**Table 1:** Implementation of nutrition improvement programs within poor counties.

	**State poverty county**	**State and province poverty county**
Implementing area in 2012	492	691
Non-implementing area in 2012	100	392
Total	592	1,083

**Table 2:** Summary statistics.

**Variable**	**Definition**	**Obs**.	**Mean**	**Std. Dev**.
Frequency of illness in the last month	Frequency of illness in the last month	9,181	0.451	0.92
Whether sick or not in the last month	1 = sick, 0 = otherwise	9,181	0.284	0.451
Children's health status	1 = children's self-rating health status is larger than 5, 0 = otherwise	2,347	0.908	0.289
Height (cm)	Children's height (cm)	8,619	115.575	27.452
Gender	1 = male, 0 = female	9,181	0.544	0.498
Household net income	Log of household net income	9,157	8.431	1.132
Water sources	1 = water sources for cooking are filtered water, 0=otherwise	9,181	0.523	0.5
Medical time	The shortest transportation time from home to medical site (min)	9,173	13.96	18.536
Medical insurance	1 = children enjoy social medical insurance, 0 = otherwise	9,085	0.472	0.499
Father's education level	Father's education level (0–6)	9,181	2.562	1.045
Mother's education level	Mother's education level (0–6)	9,181	2.284	1.066
Whether live with father or not	1 = live with father, 0 = otherwise	9,169	0.925	0.263
Whether live with mother or not	1 = live with mother, 0 = otherwise	9,137	0.933	0.25
Father's health status	Father's self-rating health status (1–5)	5,468	2.335	1.172
Mother's health status	Mother's self-rating health status (1–5)	5,914	2.489	1.249

## Baseline results

### Estimation results

We use DID approach to estimate the effect of the nutrition improvement program on children health in the baseline analysis. Children health status is measured by the frequency of illness in the last month, whether sick or not in the last month, self-rating health status etc., respectively. Table 3A reports the estimated coefficients obtained based on the full sample of rural areas. The results are realistic, the nutrition improvement program significantly decreases the frequency of monthly illness of children by 0.2 times and the probability of illness by 8.55% at the 1% level, but the effect on individual health status is not significant. Thus, overall, the nutrition improvement program significantly improves children's health status, which is consistent with the previous findings (23–25)^7^. In addition, some well-established child nutrition interventions, such as the US school nutrition programs (26) and India's midday meals program (25), have an impact on child health in the range of 5–15%. Considering that China's nutrition improvement program is still in the early stages of implementation, the impact of its health improvement effects is still significant, as it is about 2/3 of the impact of these well-established interventions.

**Table 3:** Baseline analysis: Implementing areas and non-implementing areas of nutrition improvement program.

	**(1)**	**(2)**	**(3)**
	**Frequency of illness**	**Whether sick or not (0–1)**	**Health status**
**(A) Implementing areas and non-implementing areas (all counties)**			
SNIP	−0.204***	−0.0855***	0.0258
	(0.0345)	(0.018)	(0.0364)
Observations	8,808	8,808	2,256
*R* ^2^	0.048	0.051	0.057
**(B) Implementing areas and non-implementing areas (restricted to state poverty counties)**			
SNIP	−0.208***	−0.0977***	0.0788*
	(0.0493)	(0.0246)	(0.0425)
Observations	3,797	3,797	1,016
*R* ^2^	0.063	0.066	0.085
**(C) Implementing areas and non-implementing areas (restricted to state and province poverty counties)**			
SNIP	−0.221***	−0.0859***	0.0774*
	(0.0477)	(0.023)	(0.0401)
Observations	5,763	5,763	1,529
*R* ^2^	0.053	0.055	0.07
Control variables	YES	YES	YES
District and year FE	YES	YES	YES

(i) All regressions control *X*_*ict*_, φ_*c*_, and ρ_*t*_.

(ii) Robust standard errors clustered at the district-year level are in parentheses.

(iii) ^***^p < 0.01, ^*^p < 0.1.

However, since pilot counties of the program are mainly composed of poverty counties, and economics development and sanitation infrastructure in these areas lag behind other areas, parallel trend assumption may not be satisfied. To solve this problem, we restrict samples in the control group to those in poverty counties and use DID model to estimate. In Table 3B, we compare implementing areas and non-implementing areas within state poverty counties and find that the coefficient is consistent with that in Table 3A and self-rating health status is also significantly positive. Children in poverty counties that implement the program enjoy better health condition compared with those in non-implementing poverty counties. In Table 3C, we relax the sample restriction by including both state and province poverty counties. The result in Table 3C is consistent with that in Table 3B. In general, estimates and significance level are similar when using different approaches. Therefore, the nutrition improvement program for rural compulsory education students has great achievement over the past few years, effectively improving children's health status.

Meanwhile, the coefficient in Tables 3B,C is significantly higher than that in Table 3A. The main reason is that the control group in Table 3A includes counties which are richer than poverty counties, and thus parallel trend assumption is threatened. Economic development and sanitation infrastructure in non-poverty counties are more advanced than those in poverty counties, and thus leads to children's greater health status in non-poverty counties. If non-poverty counties are included in the control group, the effect of the policy will be underestimated.

### Robustness checks

#### Permutation placebo tests

**Figure 1:**
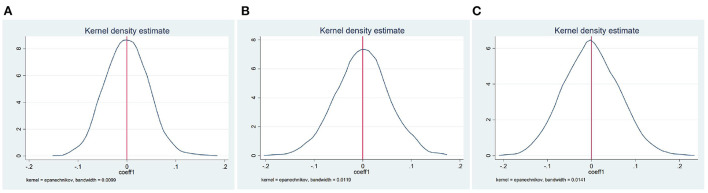
Estimated coefficients from the permutation placebo tests. **(A–C)** Show the results of permutation placebo tests for Table 3 Panels A, B, and C, respectively.

The key assumption of DID approach is parallel trend assumption. Since data before the implementation only extend for 1 year, we use bootstrap method to validate the reliability of our evaluation. Prior to the implementation of the nutrition improvement program, regional differences may have potential impacts on individual health, leading to over- or under-estimation of results in Table 3. Although we have included district fixed effects in the baseline model, there may still be other uncontrolled factors. To solve this problem, we use placebo test introduced by previous studies (27, 28), and construct dummy information at the district and year level to check robustness. Specifically, implementing areas are randomly selected. Then we construct corresponding dummy variables and regress Equation (1). Finally, we replicates this process for several times and get the distribution of the estimation of $\hat{\beta }$ coefficient. If the estimates reported in Table 3 are unbiased, dummy policy-implementation variable will not pose any impacts on fertility behavior, i.e., the mean of the estimation $\hat{\beta }$ generated by bootstrap method equals to 0 and insignificant. Figure 1 shows kernel density estimate of coefficient $\hat{\beta }$ by replicating the process above for 1,000 times. It illustrates that the estimates of $\hat{\beta }$ concentrate around 0. Therefore, the potential problem we discuss above will not affect the estimation and baseline results are robust.

#### Adding parents' health status variables

Parents' health status significantly affects children's health in the following three ways: (i) Imitation and study of living habits. Children imitate parents' habits instinctively and actively, such as daily routines, eating habits, health care habits etc. These factors can affect parents' and children's health simultaneously. (ii) Parents' health status reflects household financial situation, and household financial situation also affects children's nutrition level and access to medical care. (iii) Hereditary factors. Parents' inherited disease may negatively influence children's health. Thus, we control parents' health status variables. Since there exists much missing value of parents' health status in CFPS, parents' health is not controlled in the baseline estimation to avoid the effect of missing value on estimation. If results after adding parents' health are consistent with baseline results, the estimation must be robust.

In Table 4, columns (1), (2), and (3), we add parents' health variables as control variables, and use full samples, state poverty counties samples and both state and province poverty counties samples respectively to analyze. The absolute value of the coefficient is lowered by adding parents' health variables. Thus, these findings confirm that children's health can be partly impacted by parents' health, leading to the decline of the coefficient. Also, the coefficient is still significantly positive, which presents the robustness of baseline results.

**Table 4:** Robustness checks.

	**(1)**	**(2)**	**(3)**	**(4)**	**(5)**	**(6)**
	**Adding parents' health variables**			**Using 2010–2014 data**		
	**Frequency of illness**	**Whether sick or not (0–1)**	**Health status**	**Frequency of illness**	**Whether sick or not (0–1)**	**Health status**
**(A) Implementing areas and non-implementing areas (all counties)**						
SNIP	−0.183***	−0.0906***	0.0773	−0.148***	−0.0602***	0.0723**
	(0.0458)	(0.0238)	(0.0504)	(0.0334)	(0.0162)	(0.0337)
Observations	4,933	4,933	899	9,361	9,361	2,655
*R* ^2^	0.049	0.056	0.133	0.05	0.06	0.082
**(B) Implementing areas and non-implementing areas (restricted to state poverty counties)**						
SNIP	−0.190**	−0.0963**	0.167	−0.160***	−0.0747***	0.108*
	(0.072)	(0.0371)	(0.102)	(0.0496)	(0.0218)	(0.0555)
Observations	2,117	2,117	401	3,978	3,978	1,184
*R* ^2^	0.057	0.061	0.197	0.049	0.062	0.122
**(C) Implementing areas and non-implementing areas (restricted to state and province poverty counties)**						
SNIP	−0.175**	−0.0850**	0.160*	−0.153***	−0.0568***	0.104**
	(0.0672)	(0.0325)	(0.0893)	(0.0438)	(0.0198)	(0.0467)
Observations	3,183	3,183	613	6,022	6,022	1,755
*R* ^2^	0.058	0.061	0.155	0.049	0.057	0.094
Control variables	YES	YES	YES	YES	YES	YES
District and year FE	YES	YES	YES	YES	YES	YES

(i) All regressions control *X*_*ict*_, φ_*c*_, and ρ_*t*_.

(ii) Robust standard errors clustered at the district-year level are in parentheses.

(iii) ^***^p < 0.01, ^**^p < 0.05, ^*^p < 0.1.

#### Using 2010, 2012, and 2014 data

In the baseline analysis, we use micro panel data from 2010 to 2016. Since analysis samples focus on teenagers under 15 years old, the longer the investigation goes on, the more missing samples are generated. To enlarge the volume of analysis samples, we regress on the same model using 2010, 2012, and 2014 data. Table 4, columns (4), (5), and (6), reports that the results are all robust, with the coefficient when using self-rating health being significant in Table 4A. It further shows that nutrition improvement program significantly improves adolescent health. Also, compared with the results reported in Table 3, the absolute value of the coefficients reported in Table 4, columns (4), (5), and (6), is lowered but still statistically significant, which may be caused by the lagged effect of the policy implementation.

There has existed some studies which conclude that early nutrition interventions can have both short- and long-term effects. Early childhood interventions have highly significant short-term effects on children's physical health development, and have significant positive effects on children's cognitive development in the long term (23). Health interventions during adolescence have not only short-term effects for vulnerable populations, but also long-term positive effects on their health levels (29). Some scholars evaluates the costs and benefits of Head Start, which is the largest early childhood education program in the United States and find that early childhood interventions have significant long-term effects, with quickly declining short-term effects (30). In general, early nutrition interventions yield long-term effects, supported by the existing literature.

## Heterogeneity analysis

### Effect of geographic factors

There exists sharp differences in economic development among eastern, central, and western regions in China. All state poverty counties are located in central and western regions. Differences in economic development among regions indirectly lead to the gap in children's health status around the nation through affecting household income levels, nutrition intake, and health conditions. Thus, we analyze heterogeneous impacts of the nutrition improvement program on health status of children in eastern, central, and western regions.

Table 5 reports that the impact of the nutrition improvement program on health status of children varies greatly in eastern, central, and western regions. The impact is larger for children in western regions and a bit smaller for children in central regions, compared with those in eastern regions. The following two factors are attributed to this results. First, the nutrition improvement program will significantly improve the amount and variety of food intake in impoverished western regions. On one hand, since poorer families spend less on food, marginal utility of the nutrition improvement program on impoverished areas is larger. On the other hand, schools are responsible for purchasing food and arranging nutritious meals every day, so the price will be relatively low and there will be a wider variety of food, which poses a more positive impact on teenagers in impoverished regions, especially in remote regions. Second, there exists regional differences in sanitation infrastructure. Eastern regions in China have more abundant health care resources and higher quality of access to medical care. Thus, differences in the quality of medical care of eastern, central, and western regions can have a direct impact on children's health. Not only are children from poorer families more likely to suffer from several chronic diseases, but they are also less likely to effectively address some health issues (31). Thus, the nutrition improvement program would have a greater marginal impact on children in western regions, where medical conditions and nutrition status are relatively backward. This implies that the nutrition improvement program also narrows the gap among adolescents in western and eastern regions, which can affect the quality of human capital in the long run.

**Table 5:** Heterogeneity analysis.

	**(1)**	**(2)**	**(3)**	**(4)**	**(5)**	**(6)**	**(7)**
	**Eastern region**	**Central region**	**Western region**	**Not living with parents**	**Living with parents**	**Male**	**Female**
**(A) Frequency of illness**							
SNIP	−0.118*	−0.172***	−0.251***	−0.300***	−0.141***	−0.212***	−0.0940***
	(0.0625)	(0.0495)	(0.0535)	(0.097)	(0.0462)	(0.0381)	(0.0182)
Observations	1,839	3,195	3,774	777	8,031	4,805	4,003
*R* ^2^	0.068	0.052	0.046	0.131	0.123	0.046	0.054
**(B) Whether sick or not (0–1)**							
SNIP	−0.0157	−0.0908**	−0.100***	−0.197***	−0.0817***	−0.193***	−0.0744***
	(0.0404)	(0.033)	(0.0234)	(0.0378)	(0.019)	(0.0554)	(0.0264)
Observations	1,839	3,195	3,774	777	8,031	4,805	4,003
*R* ^2^	0.073	0.046	0.051	0.045	0.05	0.062	0.058
**(C) Children's health status**							
SNIP	0.134*	0.0136	−0.336	0.0812*	0.0176	0.028	0.0227
	(0.0688)	(0.0397)	(0.24)	(0.0481)	(0.0397)	(0.0302)	(0.0478)
Observations	483	738	1,035	211	2,045	1,206	1,050
*R* ^2^	0.084	0.1	0.041	0.134	0.057	0.079	0.061
Control variables	YES	YES	YES	YES	YES	YES	YES
District and year FE	YES	YES	YES	YES	YES	YES	YES

(i) All regressions control *X*_*ict*_, φ_*c*_, and ρ_*t*_.

(ii) Robust standard errors clustered at the district-year level are in parentheses.

(iii) ^***^p < 0.01, ^**^p < 0.05, ^*^p < 0.1.

### Effect of living with parents

As rural migrant workers go out to work, a large number of children are left behind in hometowns. Many scholars find that the lack of care caused by parents' migrant work has a negative impact on children's health (14, 32). This negative effect is positively proportional to the number of children left behind in a household, partly because children's nutrition intake may be influenced by whether living with parents or not. To identify the effect of parents' migrant work, we analyze heterogeneous impacts of the nutrition improvement program on health status of children who live with parents and who do not live with parents. Table 5 reports that the effect of policy implementation is larger for children who do not live with their parents. Thus, the nutrition improvement program can partially alleviate nutrition deficiency of children whose parents are migrant workers.

Parents' time investment in children can decisively effect children's health performance in the following two ways: (i) Feedback effect. If parents live with their children, mothers can adjust their current resource investment decisions according to children's performance, so that children's health level and other performance will be better (34). In addition, mothers' time investment can affect adolescents' cognition skills, and this effect gradually declines with the increase of children's age (35). (ii) Separation effect. If parents do not live with their children, prolonged separation can not only be detrimental to children's health, but also have a negative impact on children's education and cognitive level (36).

### Effect of gender

According to Report on Nutrition and Chronic Diseases in China (2015), the growth retardation rate and wasting rate of male children aged 6–17 years are higher than those of female children in 2012. We analyze heterogeneous impacts of the nutrition improvement program on health status of male and female children. Table 5 reports that the effect of the policy implementation is larger for boys, which is consistent with the previous literature (37). Boys benefit more from high-quality care programs than low-quality ones, and boys become more vulnerable after leaving their mothers, which do not exist among girls (38). This is mainly due to the fact that girls become mature earlier and have stronger capability to encounter adversity, so they can perform better in environments with higher pressure and lower quality (39).

## Evidence for role of nutrition diversity and physical quality

### Nutrition diversity

Food variety and nutrition diversity are key variables affecting children's health (26). Nutrition improvement program promotes food richness and diversity by providing free meals to children in impoverished areas. The school breakfast program plays a relatively important role in nutrition intake of students, which can improve intake of protein, carbohydrate, calcium, iron and other trace elements, as well as vitamin C, vitamin E, folic acid, and other nutrients (26, 40). Therefore, we assume nutrition diversity as a mechanism by which nutrition improvement program works.

Whether eight kinds of food listed in Table 6 are consumed is chosen as dependent variables, and we still use DID model to regress. Table 6 reports that policy implementation helps increase children's intake of beans and decrease their intake of preserved food. On one hand, students' diet becomes healthier through providing soy milk and other foods after the policy implementation. On the other hand, students' demand for unhealthy food, especially preserved food, dropped dramatically after providing a nutritious breakfast, which improves health status in another way. Preserved food has a large amount of salt and contains nitrite and its derivatives, which has a negative impact on health and even causes stomach cancer (41). The substitution of health food for preserved food constitutes a crucial mechanism of nutrition improvement program. In addition, the coefficients of intake of other food are all insignificant, which may be caused by small sample size because CFPS only interviews children aged from 10 to 15 years old about their intake of eight kinds of food listed in Table 6.

**Table 6:** Nutrition diversity.

	**(1)**	**(2)**	**(3)**	**(4)**	**(5)**	**(6)**	**(7)**	**(8)**
	**Meat**	**Fish**	**Vegetable**	**Milk**	**Bean**	**Egg**	**Preserved food**	**Fried food**
**(A) Implementing areas and non-implementing areas (all counties)**								
SNIP	0.0465	−0.381	−0.0321	−0.11	0.659**	−0.0424	−0.592*	−0.462
	(0.0862)	(0.359)	(0.0418)	(0.168)	(0.309)	(0.686)	(0.343)	(0.333)
Observations	1,130	1,130	1,130	1,130	1,130	1,130	1,130	1,130
*R* ^2^	0.206	0.239	0.246	0.135	0.149	0.138	0.118	0.095
**(B) Implementing areas and non-implementing areas (restricted to state poverty counties)**								
SNIP	−0.0154	−0.301	−0.000804	−0.0744	0.667***	−0.0427	−0.303	−0.286
	(0.0158)	(0.521)	(0.0167)	(0.0643)	(0.177)	(0.748)	(0.18)	(0.516)
Observations	507	507	507	507	507	507	507	507
*R* ^2^	0.19	0.129	0.207	0.163	0.159	0.16	0.128	0.117
**(C) Implementing areas and non-implementing areas (restricted to state and province poverty counties)**								
SNIP	0.0464	−0.433	−0.0496	−0.12	0.642**	0.0236	−0.652*	−0.513
	(0.0942)	(0.357)	(0.0358)	(0.178)	(0.3)	(0.694)	(0.357)	(0.367)
Observations	772	772	772	772	772	772	772	772
*R* ^2^	0.205	0.264	0.237	0.14	0.162	0.152	0.142	0.101
Control variables	YES	YES	YES	YES	YES	YES	YES	YES
District and year FE	YES	YES	YES	YES	YES	YES	YES	YES

(i) All regressions control *X*_*ict*_, φ_*c*_, and ρ_*t*_.

(ii) Robust standard errors clustered at the district-year level are in parentheses.

(iii) ^***^p < 0.01, ^**^p < 0.05, ^*^p < 0.1.

### Physical quality

The second impact mechanism is physical quality, but concensus has not been achieved among researchers. Some researchers find that the school breakfast or lunch program can keep students' body mass index (BMI) at a relatively healthy level (42). However, some researchers find that some school lunch programs lead students to overeat calories, which leads to obesity, and only a healthy and balanced meal is the optimized choice (43). We analyze this impact mechanism by selecting height, growth retardation, and excessive thinness as dependent variables, and the result is reported in Table 7.

**Table 7:** Physical quality.

	**(1)**	**(2)**	**(3)**
	**Height**	**Growth retardation**	**Excessive thinness**
**(A) Implementing areas and non-implementing areas (all counties)**			
SNIP	0.198***	−0.275***	−0.207***
	(0.0166)	(0.0233)	(0.0233)
Observations	8,277	8,498	8,465
*R* ^2^	0.53	0.348	0.314
**(B) Implementing areas and non-implementing areas (restricted to state poverty counties)**			
SNIP	0.202***	−0.266***	−0.212***
	(0.023)	(0.0283)	(0.0291)
Observations	3,508	3,644	3,627
*R* ^2^	0.561	0.357	0.341
**(C) Implementing areas and non-implementing areas (restricted to state and province poverty counties)**			
SNIP	0.211***	−0.287***	−0.228***
	(0.0203)	(0.0273)	(0.0273)
Observations	5,399	5,558	5,536
*R* ^2^	0.547	0.352	0.324
Control variables	YES	YES	YES
District and year FE	YES	YES	YES

(i) All regressions control *X*_*ict*_, φ_*c*_, and ρ_*t*_.

(ii) Robust standard errors clustered at the district-year level are in parentheses.

(iii) ^***^p < 0.01.

Table 7, column (1), takes height as the dependent variable. When using different restriction on samples, we can all conclude that the nutrition improvement program significantly increases the height of teenagers by about 0.2 cm. According data published by Chinese Association for Student Nutrition and Health Promotion, the average height of boys and girls aged from 6 to 15 years old increases by 0.4 and 0.6 cm compared with the same period last year after policy implementation.

In addition, according to height retardation criteria of all ages and sexes in National Health Standards (33), and moderate to severe weight loss defined by BMI, we define dummy variables that measure growth retardation and excessive thinness based on individual's height and BMI in each survey year. That is to say, if individual's height and BMI are below the standard, he or she will be defined as “growth retardation” and “excessive thinness.” Table 7, columns (1), (2), and (3), report that the nutrition improvement program significantly reduces the proportion of growth retardation and excessive thinness among teenagers. Pilot counties of the nutrition improvement program are mainly poverty counties, which means that physical quality of children in these areas is poor. The nutrition improvement program significantly improves children's health status through promoting their physical quality.

## Conclusion

Children's health is related to not only long-term human capital accumulation, but also poverty alleviation through education and intergenerational poverty entrenchment. We use DID model to empirically analyze the effect of the nutrition improvement program on children's health and its impact mechanism. The results show that the nutrition improvement program leads to the decrease in the frequency of illness by about 0.2 times, the decrease in the probability of illness by about 9% and the significant improvement in students' health status, which proves robust. In the heterogeneity analysis, we find that marginal effect of the implementation of the program is more significant for children in western regions and children left behind, which indicates that the nutrition improvement program can alleviate and narrow health disparities of children in central and western regions, and help compensate for the lack of parents' care caused by migrant work. In addition, the nutrition improvement program can promote students' height and reduce the probability of growth retardation and excessive thinness.

Our conclusion highlights three crucial directions for policy makers to consider: (i) Expand subsidies. As a rare financial subsidy program, the marginal utility of the nutrition improvement program for poorer households is higher, which means that the nutrition improvement program will be targeted to improve health of children in poor families, and promote intergenerational mobility of poor families. Therefore, China should further increase subsidies for the nutrition improvement program. (ii) Expand coverage. On one hand, after completion of the pilot program, China should further expand the coverage of households benefiting from this program, so that all the youth nationwide can enjoy the policy dividend. On the other hand, since preschool children's health and ability have a strong impact on lifetime performance (38), preschool children should also benefit from the program. (iii) Improve dining environment and nutrition. China should establish more complete nutrition improvement program catering standards and improve dining environment, so that funds for the nutrition improvement program can play a greater and more effective role.

In addition, due to the limited time of the implementation of the nutrition improvement program, we have not evaluated long-term costs and benefits of the program. However, the benefits of the nutrition improvement program are not only the decline of medical costs of teenagers in the short run, but also related to adult health status, future social health consumption, human capital level, even crime rate, and other variables in the long run, which affects potential for future economic growth in China. Therefore, governments should attach importance to both short-term costs and long-term benefits when implementing the nutrition improvement program.

## Data availability statement

The original contributions presented in the study are included in the article/supplementary material, further inquiries can be directed to the corresponding author/s.

## Author contributions

YL and SJ contributed to conception and design of the study. YL, XC, and CZ organized the database and performed the statistical analysis. YL and CZ wrote the first draft of the manuscript. XC and SJ wrote sections of the manuscript. All authors contributed to manuscript revision, read, and approved the submitted version.

## Funding

This research was supported by the Science Foundation of Ministry of Education of China (22YJC790069).

## Conflict of interest

Author CZ was employed by China Export and Credit Insurance Corporation. The remaining authors declare that the research was conducted in the absence of any commercial or financial relationships that could be construed as a potential conflict of interest. The handling editor declared a shared affiliation with the author(s) SJ at the time of review.

## Publisher's note

All claims expressed in this article are solely those of the authors and do not necessarily represent those of their affiliated organizations, or those of the publisher, the editors and the reviewers. Any product that may be evaluated in this article, or claim that may be made by its manufacturer, is not guaranteed or endorsed by the publisher.

## Footnotes

^1^On one hand, health behaviors such as children anemia and hypoglycemia can affect students' attentiveness and short-term academic performance (3). On the other hand, health trauma in childhood (e.g., famine) poses a negative impact on adult health and consumption structure (4) and even has a long-term negative impact on children's cognitive skills (5).

^2^Aurino et al. (13) found that the large-scale school feeding program in Ghana led to a slight improvement in the average standardized scores of students in mathematics and literacy. In particular, greater improvements were found for girls, disadvantaged children, and regions.

^3^Meng and Yamauchi (14) find that Chinese parents' migrant work can negatively affect the education and health status of children left behind.

^4^Culture of male preference in China leads to unfair allocation of resources which is more favorable to boys, thus affecting the health of children within the family (15). Family planning policies enforce limits on the number of children in a household, which can lead to greater improvements in the health of girls in one-child households (16).

^5^Medical insurance and health infrastructure can improve household budgets and resilience to risk, thereby affecting individual health, especially in rural areas. Meanwhile, it can pose a direct impact on the intergenerational transmission of health capital. Zhang (18) studies the impact of drinking water infrastructure program in rural China on individual health using data from China Health and Nutrition Survey (CHNS). The study finds that the program significantly improves children's height, weight, and individual health.

^6^Difference-in-differences (DID) method is one of the most popular research designs used to assess the causal effects of policy interventions. By comparing the average change in outcomes experienced by the treated group and the average change in outcomes experienced by the comparison group, we could estimate the average treatment effect for the treated subpopulation (ATT). Currently, a large number of studies by scholars such as Heckman et al. (20), de Chaisemartin and D'HaultfŒuille (21), and Sun and Abraham (22), and others have adopted the DID approach.

^7^Jain (24) considers that India ICDS program, which is devoted to supporting preschool children in impoverished areas, improves children's health and further reduces morbidity and mortality through providing nutrition and immunity. Berry et al. (25) found that monitoring measures for learning health interventions affect the policy effectiveness of school nutrition programs. Specifically, high-intensity monitoring significantly improved child health.
